# Exercise-Induced Irisin Decreases Inflammation and Improves NAFLD by Competitive Binding with MD2

**DOI:** 10.3390/cells10123306

**Published:** 2021-11-25

**Authors:** Weiwei Zhu, Namood E Sahar, Hafiz Muhammad Ahmad Javaid, Eun Seon Pak, Guang Liang, Yi Wang, Hunjoo Ha, Joo Young Huh

**Affiliations:** 1 College of Pharmacy, Chonnam National University, Gwangju 61186, Korea; 206033@jnu.ac.kr (W.Z.); sahar.namood@gmail.com (N.E.S.); 186681@jnu.ac.kr (H.M.A.J.); 2 Chemical Biology Research Center, School of Pharmaceutical Sciences, Wenzhou Medical University, Wenzhou 325035, China; liangguang@wmu.edu.cn (G.L.); yi.wang1122@wmu.edu.cn (Y.W.); 3 Graduate School of Pharmaceutical Sciences, College of Pharmacy, Ewha Womans University, Seoul 03760, Korea; louisa9419@gmail.com (E.S.P.); hha@ewha.ac.kr (H.H.)

**Keywords:** irisin, myokine, MD2, NAFLD, inflammasome, exercise

## Abstract

Non-alcoholic fatty liver disease (NAFLD) is a global clinical problem. The MD2-TLR4 pathway exacerbates NAFLD progression by promoting inflammation. Long-term exercise is considered to improve NAFLD but the underlying mechanism is still unclear. In this study, we examined the protective effect and molecular mechanism of exercise on high-fat diet (HFD)-induced liver injury. In an HFD-induced NAFLD mouse model, exercise training significantly decreased hepatic steatosis and fibrosis. Interestingly, exercise training blocked the binding of MD2-TLR4 and decreased the downstream inflammatory response. Irisin is a myokine that is highly expressed in response to exercise and exerts anti-inflammatory effects. We found that circulating irisin levels and muscle irisin expression were significantly increased in exercised mice, suggesting that irisin could mediate the effect of exercise on NAFLD. In vitro studies showed that irisin improved lipid metabolism, fibrosis, and inflammation in palmitic acid (PA)-stimulated AML12 cells. Moreover, binding assay results showed that irisin disturbed MD2-TLR4 complex formation by directly binding with MD2 but not TLR4, and interfered with the recognition of stimuli such as PA and lipopolysaccharide with MD2. Our study provides novel evidence that exercise-induced irisin inhibits inflammation via competitive binding with MD2 to improve NAFLD. Thus, irisin could be considered a potential therapy for NAFLD.

## 1. Introduction

Non-alcoholic fatty liver disease (NAFLD) is the most common liver disease worldwide [1]. While there are no current United States Food and Drug Administration (FDA)-approved drugs for NAFLD treatment [2], the most effective therapy to date comprises exercise and calorie restriction [3]. Therefore, exploring the role and mechanism of exercise and screening for related endogenous molecules could be helpful in the search for potent drug candidates for NAFLD treatment.

In the NAFLD spectrum, inflammation is initiated by the aberrant accumulation of lipids, which then activates hepatic fibrosis [4]. Furthermore, chronic inflammation leads to insulin resistance, a key phenomenon in NAFLD [5]. Myeloid differentiation factor 2 (MD2) is an adaptor protein that interacts with toll-like receptor 4 (TLR4) [6]. The MD2-TLR4 complex recognizes lipopolysaccharide (LPS) and recruits intracellular adaptor protein myeloid differentiation factor 88 (MyD88), which leads to the activation of mitogen-activated protein kinase (MAPK) and nuclear factor-κB (NF-κB) pathways and release of pro-inflammatory cytokines [7]. Recently, several studies showed that the MD2-TLR4 pathway was not only activated by canonical Gram-negative bacteria but also by metabolism-associated molecular patterns (MAMPs) [8], including free fatty acids (FFAs), glucose, and cholesterol, which are all present in excess in chronic metabolic diseases such as atherosclerosis [9], obesity [10], and diabetes [11]. Therefore, moderating the MD2-TLR4 pathway can benefit metabolic diseases including NAFLD by decreasing inflammation [12].

Regular exercise enhances muscle strength, regulates blood glucose and lipid levels, and reduces the risk of metabolic diseases [13,14]. Recent reports have suggested that exercise can also suppress chronic inflammation [15]. In high-fat diet (HFD)-induced obese mice, exercise can switch the polarization of adipose tissue from the M1 to the M2 phenotype [16,17]. It has also been shown that exercise can reduce the expression of TLRs and inflammatory signaling [17,18]. However, the underlying molecular mechanism remains unclear. Myokines released by contractile muscles regulate metabolism in muscles as well as in distant organs [19]. Among the myokines discovered to date, irisin is a cleavage product of its precursor fibronectin type III domain-containing 5 (FNDC5) [20]. Exercise-induced secretion of irisin mediates the beneficial effects of exercise such as promoting adipocyte browning through uncoupling protein 1 (UCP-1) [21], facilitating glycogen synthesis to improve insulin resistance [22], and promoting skeletal remodeling through the integrin receptor [23]. Moreover, irisin was recently discovered to exert anti-inflammatory properties by suppressing the TLR4 pathway [24,25], which makes irisin the most likely effector of exercise to decrease liver inflammation.

In the present study, we investigated the effect of exercise training in HFD-induced NAFLD mice and dissected the role of exercise in MD2-TLR4 complex formation. Additional in vitro studies evaluated the mechanism by which irisin blocked MD2-TLR4 complex formation. Our findings demonstrate a novel mechanism in which prolonged endurance exercise improves NAFLD and suggest that exercise-induced irisin could interact with MD2 to exert anti-inflammatory effects in hepatocytes, highlighting its potential as a therapeutic target for treatment of NAFLD.

## 2. Materials and Methods

### 2.1. Animal Studies

Male C57BL/6 mice were obtained from Orientbio (Seongnam, South Korea). All animals were housed in a pathogen-free room at 22 ± 2 °C, 50–60% humidity, and a 12:12 h light–dark cycle. Water and food were provided ad libitum throughout the study period. The mice were randomly divided into three groups (*n* = 6 for each group): (1) normal chow diet (NCD); (2) high-fat diet (HFD); and (3) high-fat diet + exercise (HFDE). The mice were fed a high-fat diet (HFD; 60% fat; Research Diets; New Brunswick, NJ, USA) from 7 weeks old until the end of the study to induce NAFLD [26]. Treadmill exercise was conducted 5 days/week from 13 weeks old and lasted for 8 weeks. The exercise training was performed at 12–20 m/min for 20–50 min/day (70% VO2 max). The training time and speed gradually increased with time. All mice were euthanized at 21 weeks old. For plasma, blood was collected and centrifuged for 15 min at 3000 rpm at 4 °C. Liver and soleus, gastrocnemius, and quadricep muscles were collected for subsequent analysis. An Aminotransferase Activity Assay Kit (AST; Abcam, Cambridge, UK) and an Alanine Transaminase Activity Assay Kit (ALT; Abcam) were used to measure liver AST and ALT levels. All animal experiments were performed in accordance with the Laboratory Animals Welfare Act, Guide for the Care and Use of Laboratory Animals, and approved by the Institutional Animal Care and Use Committee (IACUC) at Chonnam National University.

### 2.2. Histological Staining and Scoring

Liver tissues were fixed in 10% formalin solution, embedded in paraffin, and sectioned at 5 μm thicknesses. The sections were stained with hematoxylin and eosin (H&E; Agilent, Santa Clara, CA, USA) and Sirius Red (Sigma; St. Louis, MO, USA). Immunohistochemistry for macrophage was performed using F4/80 antibody (1:200; Abcam, Cambridge, UK) and immunoreactivity was detected by diaminobenzidine (DAB). The stained sections were viewed under a light microscope (Carl Zeiss AG; Oberkochen, Germany). The H&E staining results were evaluated by the NASH-Clinical Research Network (CRN) criteria [27]. Specifically, the amount of steatosis (percentage of hepatocytes containing fat droplets) was scored as 0 (<5%), 1 (5–33%), 2 (>33–66%), and 3 (>66%). Hepatocyte ballooning was classified as 0 (none), 1 (few), or 2 (many cells/prominent ballooning). Foci of lobular inflammation were scored as 0 (no foci), 1 (<2 foci per 200× field), 2 (2–4 foci per 200× field), and 3 (>4 foci per 200× field). The NAFLD activity score (NAS) was computed from the grade of steatosis, inflammation, and ballooning. Sirius Red and F4/80-positive area were quantified by ImageJ version 1.53i (NIH). Six fields of each group in the different stains were selected for statistical analysis.

### 2.3. Cell Culture

The mouse hepatocyte AML12 cell line (ATCC; Manassas, VA, USA) was cultured in high-glucose Dulbecco’s modified Eagle medium (DMEM; HyClone, Logan, UT, USA) with 10% fetal bovine serum (FBS; HyClone), 1% penicillin/streptomycin (Invitrogen; Carlsbad, CA, USA), ITS supplement (Sigma), and 40 ng/mL dexamethasone. The mouse macrophage RAW264.7 cell line was cultured in DMEM medium with 10% FBS and 1% penicillin/streptomycin. All cells were grown in an incubator maintained at 37 °C with 5% CO_2_. Recombinant irisin was purchased from Phoenix Pharmaceuticals (Burlingame, CA, USA) and recombinant human MD2 (rhMD2) was purchased from R&D Systems (Minneapolis, MN, USA). Lipid accumulation in the hepatocytes was observed by Oil Red O staining as described previously [28].

### 2.4. Preparation of BSA-Palmitate Conjugates

To make 0.1 M sodium palmitate (PA) solution, PA (0.0278 g; Sigma) was melted in 1 mL of 0.1 M NaOH solution at 70 °C. Low-endotoxin bovine serum albumin (BSA; 1.6 g; Sigma) was dissolved in 19 mL of DMEM or DMEM/F-12 medium with gentle shaking in a water bath at 37 °C for 1 h. The PA solution was added to the continuously vortexed BSA solution drop-by-drop. Then, the mixed solution was incubated in a water bath at 37 °C for another 30 min. The vehicle control was obtained by preparing an 8% BSA solution in DMEM or DMEM/F-12 medium. Both the BSA-PA solution and the vehicle control solution were filtered through a 0.22 μm syringe filter and stored at −20 °C until further study. The approximate BSA:PA molar ratio was 1:4.

### 2.5. Western Blots and Immunoprecipitation

Cells or tissues were homogenized in RIPA buffer (Thermo Fisher Scientific; Carlsbad, CA, USA) and the protein concentration was measured using a Pierce BCA Protein Assay Kit (Thermo Fisher Scientific). Protein lysates were separated by SDS-PAGE and transferred to a PVDF membrane. The membrane was blocked with skimmed milk and incubated with primary antibodies at 4 °C overnight. Secondary antibodies were applied for 1 h at room temperature. The antibodies used are listed in Supplementary Table S1. Blot intensity was visualized by a LAS-4000 (Fuji Photo Film; Tokyo, Japan) and quantified using ImageJ. The values were normalized to housekeeping proteins.

For co-immunoprecipitation, protein lysates (100–500 μg) were incubated with precipitating antibody at 4 °C overnight, then immunoprecipitated with protein A/G agarose beads (Santa Cruz) and shaken at room temperature for 2 h. The bead-protein complexes were collected by centrifugation, washed 3 times with phosphate-buffered saline (PBS), dissociated by heating, and the target protein was analyzed by Western blots with the corresponding antibody.

### 2.6. Gene Expression Analysis

Total RNA was extracted using Trizol Reagent (MRC; Cincinnati, OH, USA). Real-time polymerase chain reaction (PCR) analysis was conducted as described previously [29]. The primers are listed in Supplementary Table S2.

### 2.7. Enzyme-Linked Immunosorbent Assay

Mouse IL-1 beta/IL-1F2 Quantikine ELISA Kit and Human Irisin/FNDC5 DuoSet ELISA purchased from R&D Systems were used to determine liver IL-1β and irisin levels in the liver, skeletal muscle, and plasma according to the manufacturer’s instructions. To determine the binding ability of irisin, the following modified methods were used.

(i) Binding ability of irisin to MD2 or TLR4: IgG, anti-MD2, and anti-TLR4 were coated separately on 96-well plates at 4 °C overnight. After blocking with BSA for 1 h, liver lysates (200 μg/mL) or rhMD2 were added and incubated for 2 h at room temperature. After washing, recombinant irisin was added and incubated for another 2 h at room temperature. The plates were washed 3 times and incubated with a biotinylated irisin antibody for 2 h at room temperature. Subsequently, horseradish peroxidase (HRP), TMB substrate, and 2N HCl solution were used following conventional ELISA methods.

(ii) Effect of irisin on the formation of the MD2-TLR4 complex in the liver: 96-well plates were coated with anti-TLR4, then incubated with liver lysates (200 μg/mL). The rhMD2 solution and irisin at graded concentrations were mixed prior to adding to the plate for 2 h at room temperature. The plates were washed and then incubated with anti-MD2 and an HRP-conjugated secondary antibody. Colorimetric analysis was performed as described above.

(iii) Competition of irisin with LPS or PA for binding to MD2: BSA conjugated-LPS (0.5 mg/mL; Sigma) or -PA (5 mM) were coated onto 96-well plates. The rhMD2 solution with different concentrations of irisin was added and detected with anti-MD2 as described above.

(iv) Amount of irisin-MD2 complex in the liver: 96-well plates were coated with anti-MD2, then incubated with liver lysates (200 μg/mL). The plates were washed 3 times and detected with a biotinylated irisin antibody.

In all ELISA experiments, the OD value at 450 nm was normalized to the value at OD 570 nm. Where appropriate, the normalized values were further corrected using the total protein concentrations.

### 2.8. Surface Plasmon Resonance Assay

The binding affinity of irisin with rhMD2 was determined using a Biacore T200 instrument (Cytiva; Marlborough, MA, USA) with a CM5 sensor chip (Cytiva). Briefly, a target protein was loaded onto the sensors using an Amine Coupling Kit (Cytiva). The irisin samples were prepared with running buffer (PBS, 0.5% P20). Sensor and sample plates were placed on the instrument, and the irisin samples were flowed over the black and target sensors. Seven concentrations were injected successively at a flow rate of 30 μL/min for a 200 s association phase, which was followed by a 200 s dissociation phase at 25 °C. The final graphs were obtained by subtracting blank sensorgrams and blank samples from the duplex. The data were analyzed with Biacore T200 software EV. The dissociation constant (KD) was calculated by global fitting of the kinetics data from various concentrations of irisin using a 1:1 Langmuir binding model.

### 2.9. Protein–Protein Docking

The crystal structures of irisin (PDB: 4LSD) and human MD2 (PDB: 2E56) were derived from the RCSB Protein Data Bank (https://www.rcsb.org/ accessed on 5 June 2021). The ClusPro server 2.0 (https://www.cluspro.org/ accessed on 5 June 2021) was used to perform protein–protein docking analysis according to the published protocol [30]. The optimal conformation in rank by cluster size was chosen to further analyze and calculate the interface residues and interface hydrogen bonds using PyMOL software version 2.5.

### 2.10. Statistical Analysis

All data are reported as the mean ± SEM. Statistical analyses were performed with GraphPad Prism 8.0 software (San Diego, CA, USA). We used one-way ANOVA followed by Dunnett’s post hoc test when comparing more than two groups of data and one-way ANOVA and the nonparametric Kruskal–Wallis test, followed by Dunnett’s post hoc test, when comparing multiple independent groups. *p*-values of 0.05 were considered statistically significant. Post-tests were run only if F achieved a *p*-value of <0.05 and there was no significant variance in homogeneity.

## 3. Results

### 3.1. Exercise Improves Liver Injury in HFD-Induced NAFLD Mice

**Figure 1:**
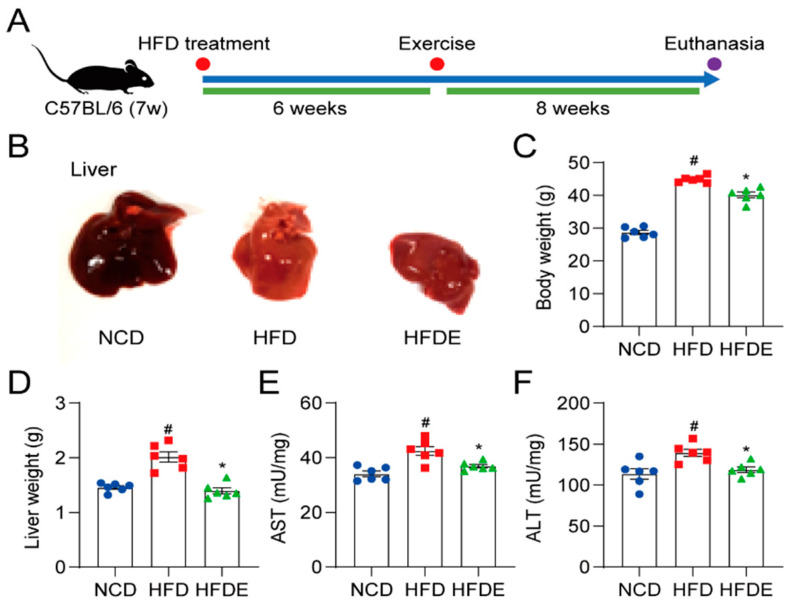
Liver gross appearance, body weight, liver weight, and liver enzymes in mice. (**A**) The NAFLD experimental C57BL/6 mouse model was generated according to the schematic timeline. (**B**) After sacrifice, the gross liver appearance was imaged. (**C**,**D**) The body weight (**C**), as well as the liver weight (**D**) was recorded. (**E**,**F**) Liver damage was assessed by measuring the hepatic levels of AST (**E**) and ALT (**F**). The data are presented as the mean ± SEM, *n* = 6 per group. # *p* < 0.05 vs. NCD group; * *p* < 0.05 vs. HFD group.

Exercise is vital for the resolution of NAFLD [3]. To clarify the underlying mechanisms, we used the HFD-induced NAFLD mouse model and performed treadmill exercise training (Figure 1A). As expected, the HFD livers showed a lighter color due to fat accumulation, which indicated the development of NAFLD. Exercise training reduced the HFD-induced changes in liver morphology (Figure 1B). HFD alone significantly increased body weight by about 50%, which was significantly reduced by exercise (Figure 1C). In addition, liver weight was increased by HFD, which was completely prevented in the exercise group (Figure 1D). Similarly, liver AST and ALT levels were induced by HFD and were significantly improved in the exercise group (Figure 1E,F). These results prove that exercise ameliorated overweight status and liver injury in NAFLD mice.

### 3.2. Exercise Protects against Steatosis and Fibrosis in NAFLD Mouse Liver

**Figure 2:**
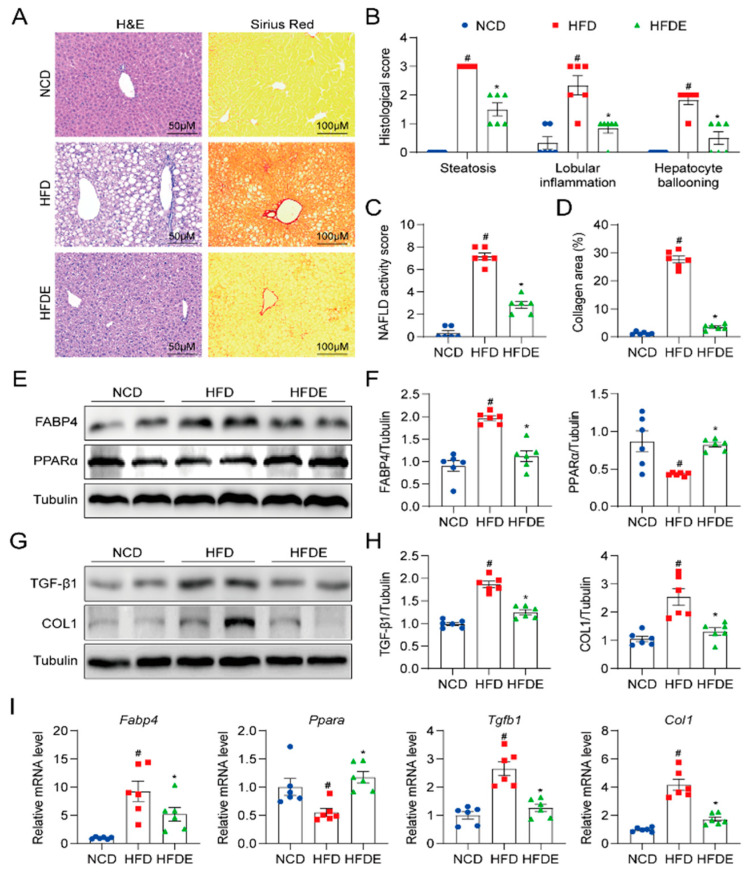
Exercise decreases steatosis and fibrosis in mouse livers. (**A**) Representative images of liver sections stained by H&E (200×, left panel) and Sirius Red (100×, right panel). (**B**) Histological scores of steatosis, lobular inflammation, and hepatocyte ballooning in H&E-stained livers. (**C**) NAFLD activity score was calculated by the histological score of steatosis, lobular inflammation, and hepatocyte ballooning. (**D**) The collagen areas seen by Sirius Red staining. (**E**,**F**) Protein levels of lipid metabolism markers FABP4 and PPARα in mouse liver tissues. Tubulin was used as the loading control. (**G**,**H**) Protein levels of fibrosis markers TGF-β1 and COL1 in mouse liver tissues. Tubulin was used as the loading control. (**I**) Relative mRNA levels of Fabp4, Ppara, Tgfb1, and Col1 in mouse liver tissues. The data are presented as the mean ± SEM, *n* = 6 per group. # *p* < 0.05 vs. NCD group; * *p* < 0.05 vs. HFD group.

Steatosis and fibrosis are two pathological processes in NAFLD [31]. H&E and Sirius Red staining were performed for liver histopathology and fibrosis assessment (Figure 2A and Supplementary Figure S1A,B). Through H&E staining, NAFLD activity was evaluated by three observations: steatosis, lobular inflammation, and hepatocyte ballooning (Figure 2B). Compared to NCD, a large number of lipid droplets were observed in the HFD group, which indicated macrovesicular steatosis, and the HFDE group showed a significant improvement of steatosis in the liver. Lobular inflammation and hepatocyte ballooning were both plainly visible in HFD-fed mice and were improved by exercise training. With a total of 6 different fields chosen, the NAFLD activity score was calculated and proved that NAFLD was partly prevented by exercise (Figure 2C). In addition, the Sirius Red quantification results showed that collagen was largely deposited in the liver by HFD treatment and eliminated by exercise (Figure 2D). Further analysis of the liver lysates showed increases in the lipid metabolism marker fatty acid-binding protein 4 (FABP4) (Figure 2E,F) and fibrogenesis markers transforming growth factor-beta 1 (TGF-β1) and collagen type I (COL1) (Figure 2G,H) in the HFD group. In contrast, peroxisome proliferator-activated receptor alpha (PPARα), which regulates fatty acid oxidation, was significantly decreased (Figure 2E,F). The exercise training results showed complete normalization of these proteins. Consistent with these results, the mRNA levels of FABP4, PPARα, TGF-β1, and COL1 showed the same trend as the protein levels (Figure 2I). These results prove that exercise effectively improved lipid metabolism and inhibited fibrogenesis.

### 3.3. Exercise Attenuates Inflammation Response in NAFLD Mouse Liver by Blocking the MD2-TLR4 Pathway

**Figure 3:**
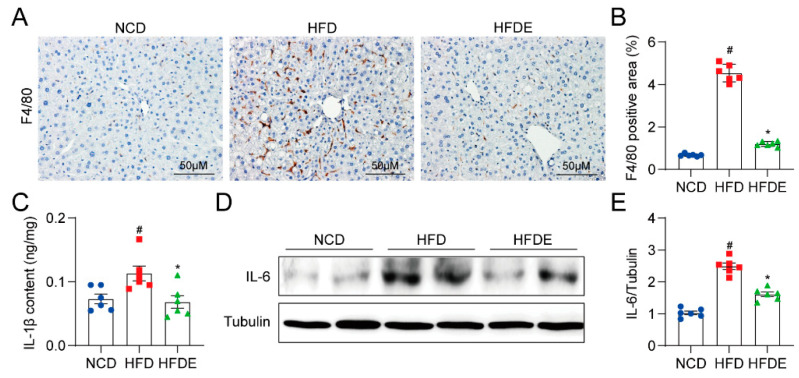
Exercise decreases inflammation in mouse livers. (**A**) Representative F4/80 immunohistochemical staining of liver sections (200×). (**B**) Quantification of F4/80-positive area in immunohistochemical staining. (**C**) Cytokine levels of inflammation marker IL-1β in mouse liver tissues. (**D**,**E**) Protein levels of inflammation marker IL-6 in mouse liver tissues. Tubulin was used as the loading control. The data are presented as the mean ± SEM, *n* = 6 per group. # *p* < 0.05 vs. NCD group; * *p* < 0.05 vs. HFD group.

Inflammation plays a key role in the transition of steatosis to fibrosis and thus, in promoting NAFLD progression [4]. To determine whether exercise regulated the inflammatory response in NAFLD mice, we first explored macrophage infiltration by F4/80 staining (Figure 3A and Supplementary Figure S1C). The results show that HFD increased the F4/80-positive areas in the liver, whereas the mice with exercise training showed marked reductions (Figure 3B). Moreover, the protein levels of pro-inflammatory cytokines interleukin-1 beta (IL-1β) (Figure 3C) and interleukin-6 (IL-6) (Figure 3D,E) were upregulated by HFD feeding. Exercise restrained IL-1β and IL-6 levels similar to those observed in the NCD mice. These results suggest that exercise limited the pathological development of NAFLD by weakening the inflammatory response.

Considering that IL-1β and IL-6 exhibited changes from exercise training, we examined whether the underlying mechanism was related to the NLR family pyrin domain containing 3 (NLRP3) inflammasome pathway. We found that the NLRP3 expression was triggered by HFD and reduced by exercise, but HFD had no effect on the expression of caspase-1 or cleavage of gasdermin D (GSDMD) (Supplementary Figure S2), indicating that exercise may be involved in the regulation of NLRP3 inflammasome assembly but not in the downstream activation pathway in the liver.

**Figure 4:**
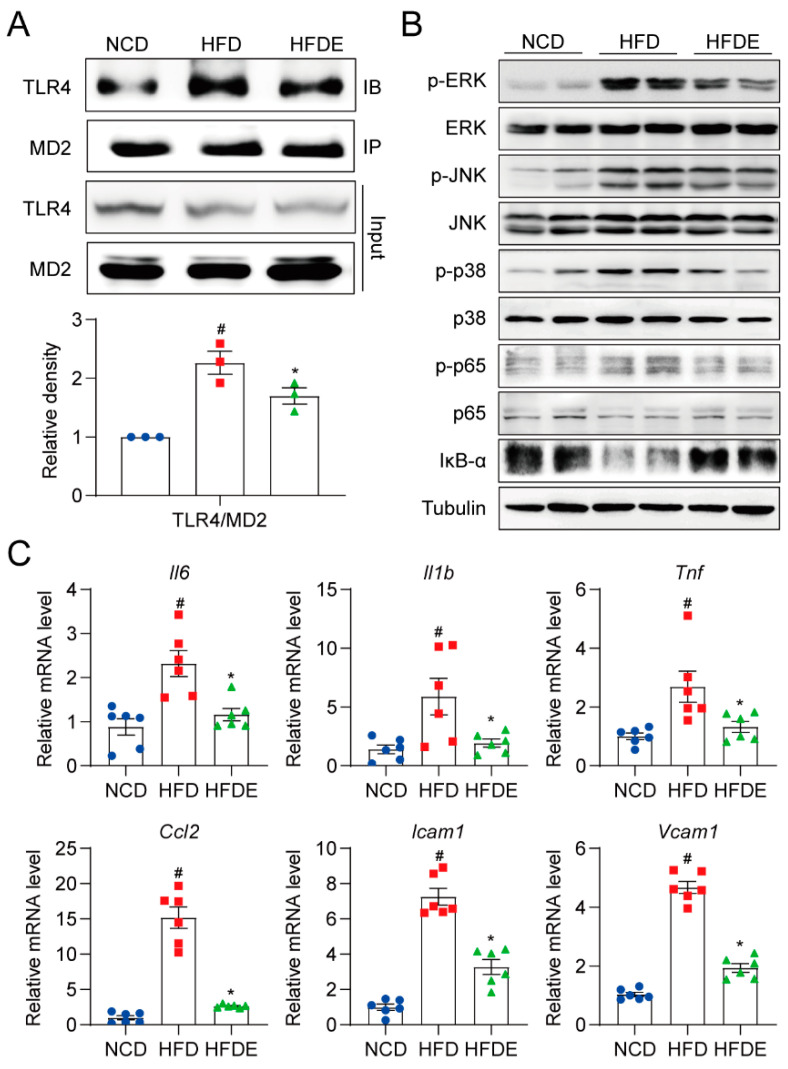
Exercise blocks MD2-TLR4 pathway activation in mouse livers. (**A**) MD2-TLR4 complex formation levels in mouse liver tissues detected by co-immunoprecipitation. (**B**) Protein levels of MAPK pathway and NF-κB pathway components, including p-ERK, p-JNK, p-p38, p-p65, and IκB-α. The corresponding unphosphorylated proteins and tubulin were used as the loading controls. (**C**) Relative mRNA levels of several pro-inflammatory markers Il6, Il1b, Tnf, Ccl2, Icam1, and Vcam1 in mouse liver tissues. The data are presented as the mean ± SEM, *n* = 6 per group. # *p* < 0.05 vs. NCD group; * *p* < 0.05 vs. HFD group.

To further investigate the underlying mechanisms, we focused on the MD2-TLR4 pathway, which contributes to the inflammatory response in NAFLD [4]. Despite the fact that the mRNA levels of MD2 and TLR4 were both increased by HFD and repressed by exercise (Supplementary Figure S3A), the protein levels of MD2 and TLR4 unexpectedly were not altered in any of the groups (Supplementary Figure S3B,C). We then evaluated whether the MD2-TLR4 complex formation was affected. The immunoprecipitation results showed that HFD highly activated MD2-TLR4 complex formation, which was strongly inhibited by exercise (Figure 4A). Consequently, the TLR4-mediated downstream MAPK and NF-κB signaling pathways in the liver showed corresponding trends. In HFD mouse livers, the phosphorylation of ERK, JNK, p38 MAPK was significantly increased while the NF-κB pathway was activated as illustrated by the increased phosphorylation of p65 and decreased levels of IκB-α, and exercise reversed these HFD-induced signaling activations (Figure 4B and Supplementary Figure S4A). In addition, the mRNA levels of inflammatory genes regulated by the TLR4 pathway (Il6, Il1b, Tnf, Ccl2, Icam1, and Vcam1) were all increased by HFD and reduced by exercise (Figure 4C). These results imply that exercise inhibited inflammation by blocking the MD2-TLR4 interaction, without changing the protein expression levels of MD2 and TLR4.

### 3.4. Irisin Is Highly Expressed in Skeletal Muscle and Blood Circulation after Exercise Training

**Figure 5:**
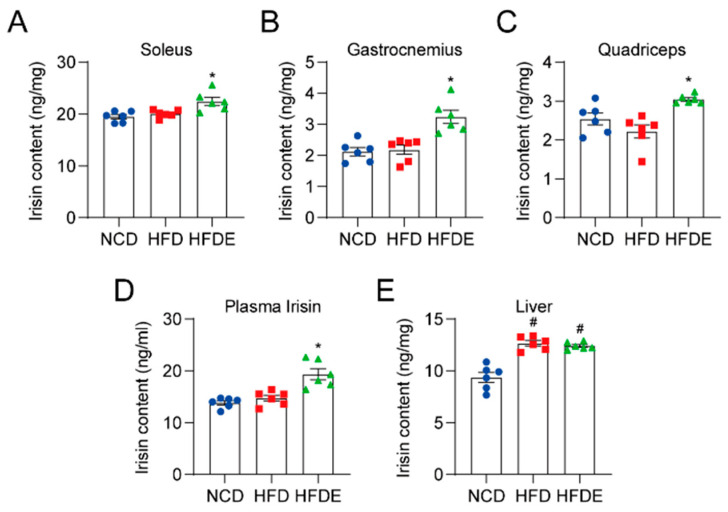
Irisin changes in several tissues and circulating levels. Absolute irisin content in several skeletal muscles including the soleus (**A**), gastrocnemius (**B**), and quadriceps (**C**), circulating levels of irisin (**D**), and absolute hepatic irisin content (**E**) in mice were measured by ELISA. The data are presented as the mean ± SEM, *n* = 6 per group. # *p* < 0.05 vs. NCD group; * *p* < 0.05 vs. HFD group.

Irisin was previously reported to exert anti-inflammatory effects in LPS-stimulated liver injury [32]. Hence, we hypothesized that irisin could mediate the anti-inflammatory role of exercise in regulating HFD-induced NAFLD. We first analyzed irisin levels in various types of muscle. As expected, irisin content in the soleus, gastrocnemius, and quadriceps muscles was increased after exercise (Figure 5A–C). Consistently, the amount of irisin in the blood was increased in the HFDE group (Figure 5D). Studies have reported that in addition to skeletal muscle, irisin could be expressed in the liver and function in an autocrine or paracrine manner [33]. As shown in Figure 5E, the hepatic irisin content was increased in the HFD and HFDE groups. Collectively, these data confirm that irisin was highly responsive to exercise training.

### 3.5. Irisin Improves Lipid Metabolism and Fibrosis in PA-Treated Hepatocytes

**Figure 6:**
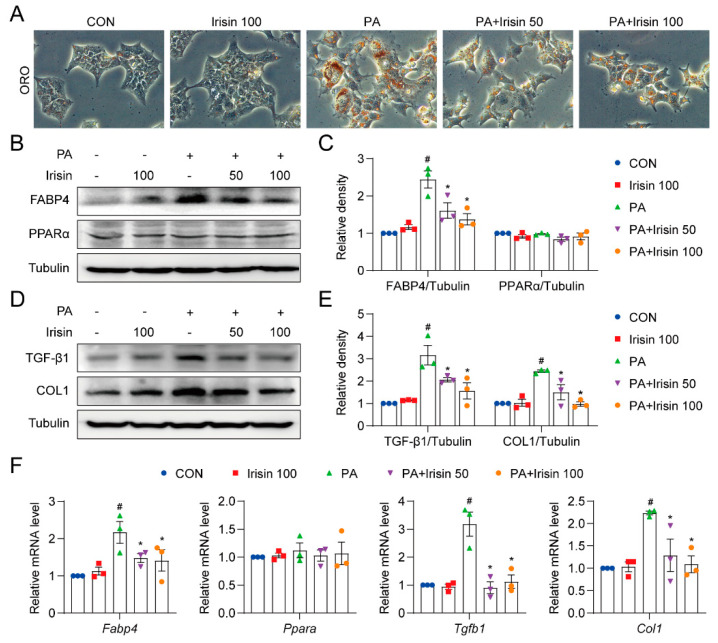
Irisin decreases steatosis and fibrosis in AML12 cells. (**A**–**C**) AML12 cells were pretreated with recombinant irisin (50 or 100 ng/mL) for 30 min followed by exposure to 200 μM PA for 36 h. (**A**) Representative images of AML12 stained by Oil Red O (400×). (**B**,**C**) Protein levels of FABP4 and PPARα in AML12 cells. (**D**,**E**) Protein levels of TGF-β1 and COL1 in AML12 cells. Tubulin was used as the loading control. (**F**) AML12 cells were pretreated with recombinant irisin (50 or 100 ng/mL) for 30 min followed by exposure to 200 μM PA for 12 h. Relative mRNA levels of Fabp4, Ppara, Tgfb1, and Col1 were detected. The data are presented as the mean ± SEM. # *p* < 0.05 vs. CON group; * *p* < 0.05 vs. PA group.

We then assessed the effect of irisin in hepatocytes. Oil Red O staining showed that irisin suppressed PA-induced lipid accumulation in AML12 cells (Figure 6A). Next, the metabolic markers measured in HFD mouse livers were also measured in hepatocytes. PA-induced FABP4 protein expression was ameliorated by irisin treatment, whereas PPARα levels were unaltered among groups (Figure 6B,C). The mRNA levels of FABP4 and PPAR**α** exhibited a similar trend as the protein levels (Figure 6F). COL1 and TGF-β1 were obviously upregulated by PA stimulation and attenuated by irisin pretreatment (Figure 6D,E). PA also stimulated the gene expression of COL1 and TGF-β1, which was suppressed by irisin pre-treatment (Figure 6F). These data confirm that irisin ameliorated lipid metabolism and fibrosis in vitro, consistent with the protective effect of irisin in hepatocytes reported by other groups [34].

### 3.6. Irisin Inhibits Inflammation by Blocking Aggregation of the MD2-TLR4 Complex and Its Predominant Pathways in PA-Treated Hepatocytes

**Figure 7:**
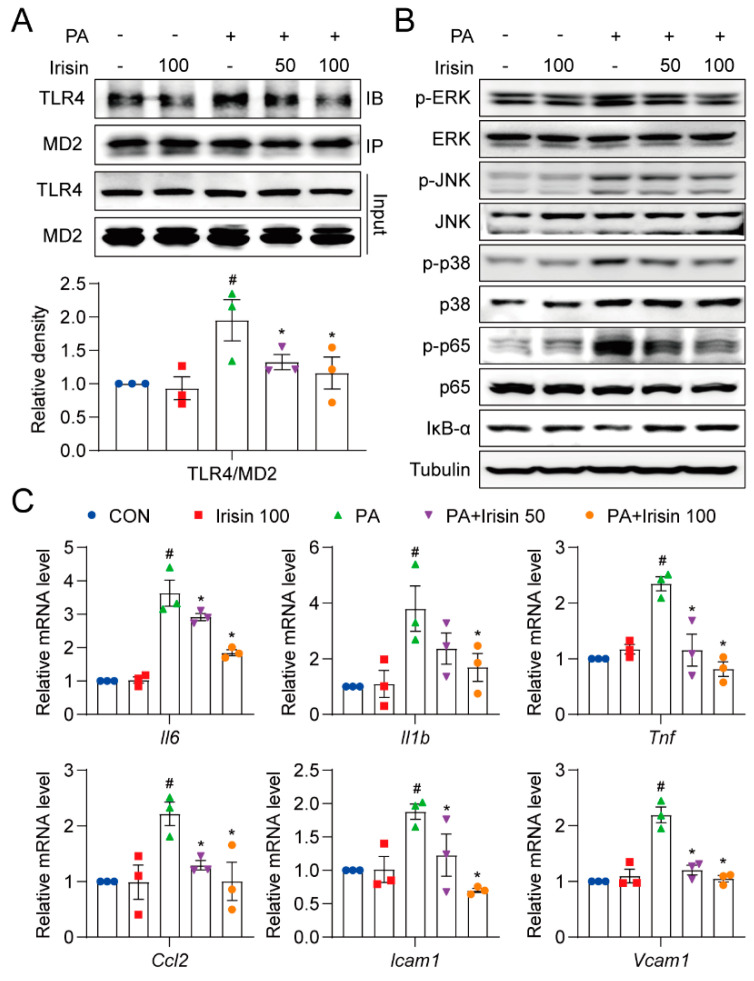
Irisin blocks NF-κB and MAPK pathways, and reduces inflammatory factors in AML12 cells. (**A**,**B**) AML12 cells were pretreated with recombinant irisin (50 or 100 ng/mL) for 30 min followed by exposure to 200 μM PA for 2 h. (**A**) MD2-TLR4 complex formation levels in AML12 cells detected by immunoprecipitation. (**B**) Protein levels of MAPK pathway and NF-κB pathway components, including p-ERK, p-JNK, p-p38, p-p65, and IκB-α. The corresponding unphosphorylated proteins and tubulin were used as loading controls. (**C**) AML12 cells were pretreated with recombinant irisin (50 or 100 ng/mL) for 30 min followed by exposure to 200 μM PA for 12 h. Relative mRNA levels of Il6, Il1b, Tnf, Ccl2, Icam1, and Vcam1 were detected. The data are presented as the mean ± SEM. # *p* < 0.05 vs. CON group; * *p* < 0.05 vs. PA group.

To confirm whether irisin exerted its anti-inflammatory effect through the MD2-TLR4 pathway, similar to the effect of exercise observed in vivo, immunoprecipitation was performed to measure the MD2-TLR4 complex. The results showed that PA treatment induced the formation of the MD2-TLR4 complex, which was suppressed by recombinant irisin treatment (Figure 7A). ERK, JNK, p38, and p65 phosphorylation and the degradation of IκB-α were increased by PA challenge, whereas irisin dose-dependently prevented the increases (Figure 7B and Supplementary Figure S4B). Furthermore, target genes including Il6, Il1b, Tnf, Ccl2, Icam1, and Vcam1 were all abundant in the PA-treated group and downregulated by irisin pre-treatment (Figure 7C). These results indicate that irisin interfered with the binding of the MD2-TLR4 complex and downstream signaling cascade, representing anti-inflammatory activity.

Monocyte-derived macrophages mainly contribute to the progression of inflammatory conditions [35]. Since our results also show the increased infiltration of immune cells in mouse liver (Figure 3A), we evaluated the anti-inflammatory effect of irisin in RAW264.7 cells. The inhibitory effect of irisin was confirmed by the normalization of PA-induced NF-κB activation and phosphorylation of p65, ERK, JNK, and p38 MAPK (Supplementary Figure S5A,B). The gene expression levels of Il6, Il1b, Tnf, Ccl2, Icam1, and Vcam1 were consistent with the results in AML12 cells (Supplementary Figure S5C). These results indicated that the anti-inflammatory role of exercise in the liver may partly be due to the inhibitory effect of irisin in macrophages.

### 3.7. Irisin Directly Binds to MD2 but Not TLR4

**Figure 8:**
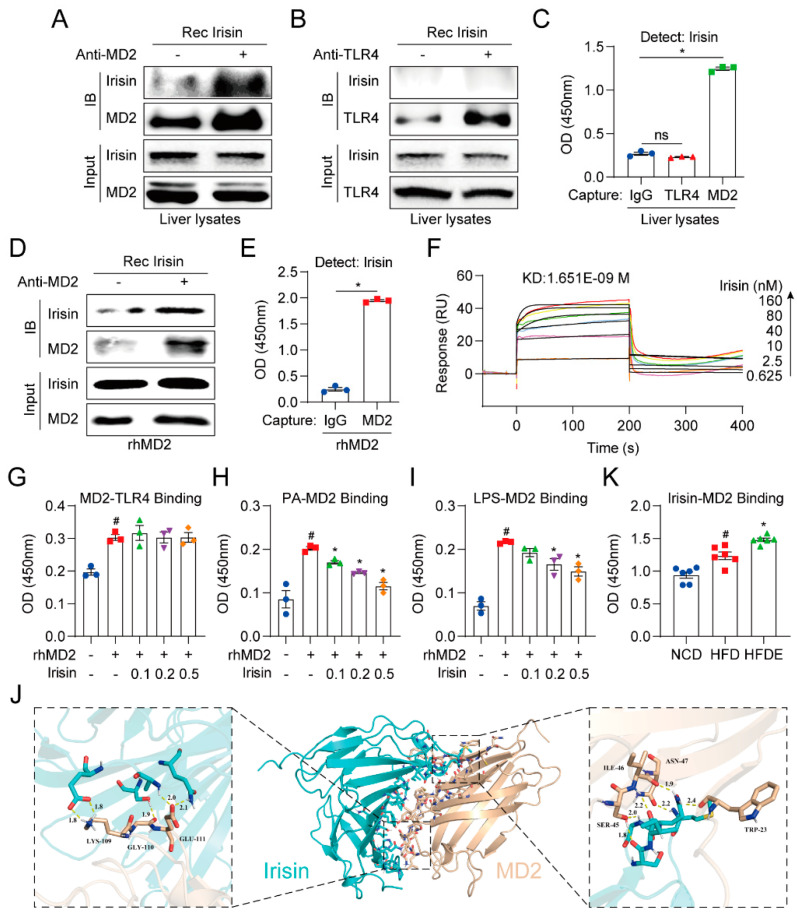
Irisin competitively binds to MD2 but not TLR4. (**A**,**B**) Immunoprecipitation analysis of the binding ability of recombinant irisin to MD2 (**A**) or TLR4 (**B**) in liver lysates. (**C**) ELISA analysis in the binding ability of recombinant irisin to MD2 or TLR4 in liver lysates. (**D**) Immunoprecipitation analysis in the binding ability of recombinant irisin to rhMD2. (**E**) ELISA analysis of the binding ability of recombinant irisin to rhMD2. (**F**) Surface plasmon resonance analysis between irisin with rhMD2. (**G**) ELISA analysis of the effect of recombinant irisin (0.1, 0.2, and 0.5 μg/mL) on the basal binding level of MD2-TLR4. (**H**,**I**) ELISA analysis of the competitive MD2 binding ability of recombinant irisin (0.1, 0.2, and 0.5 μg/mL) to PA or LPS. (**J**) Molecular docking of the dimeric irisin-MD2 complex. (**K**) ELISA analysis of irisin-MD2 binding levels in mouse liver tissue (*n* = 6 per group). The data are presented as the mean ± SEM. # *p* < 0.05 vs. CON or NCD group; * *p* < 0.05 vs. rhMD2 or HFD group.

Since irisin interfered with MD2-TLR4 complex formation (Figure 7A), we presumed that irisin may directly bind with MD2 or TLR4. Immunoprecipitation analysis in liver lysates demonstrated that recombinant irisin bound to MD2 but not TLR4 (Figure 8A,B). In addition, we confirmed these results by utilizing an ELISA-based method, which showed high OD values in the anti-MD2 group but no change in the anti-TLR4 group compared with background (Figure 8C). More directly, further analysis using rhMD2 confirmed binding between recombinant irisin and rhMD2 (Figure 8D,E). Surface plasmon resonance analysis revealed the high binding affinity (KD = 1.651 × 10^−9^ M) and fast association/dissociation processes between irisin and MD2 (Figure 8F).

Irisin binding to MD2 could have an influence in two different ways. One is by interfering with MD2-TLR4 binding and another is by interfering with MD2 binding to upstream stimulators such as LPS or PA. To distinguish the mechanism of irisin, we employed a modified ELISA method, where the OD value represented the binding ability. As shown in Figure 8G, the affinity of MD2 binding to TLR4 was not affected as no significant changes were observed with or without irisin treatment. Next, the effect of irisin on MD2 binding with PA or LPS was evaluated. The results in the control group show that rhMD2 could anchor by binding with PA or LPS, and thus be specifically recognized by the MD2 antibody and show high OD values (Figure 8H,I). Subsequently, treatment with irisin dose-dependently decreased the OD values in both the PA-MD2 binding assay and the LPS-MD2 binding assay, indicating that irisin competed with both of these stimuli to bind with MD2. Next, conformational simulation of the irisin-MD2 complex was performed by molecular docking. Notably, the only reported crystal structure of irisin is presented as a dimer [36]. The results show two motifs for MD2, KGE (residues 109–111) and SIN (residues 45–47), which formed multiple hydrogen bonds with different chains of the irisin dimer to contribute to the interaction between irisin and MD2 (Figure 8J). We finally confirmed in mouse liver that the HFDE group showed the highest levels of the irisin-MD2 complex (Figure 8K), which would indicate the lowest activity of the MD2-TLR4 pathway in the HFDE group, and proved that irisin is a mediator of exercise benefits in NAFLD mice.

## 4. Discussion

In this study, we aimed to investigate the protective mechanism of exercise in NAFLD. The main findings are that (i) exercise and irisin exerted anti-inflammatory effects in the liver and hepatocytes, respectively; (ii) exercise induced circulating irisin as well as irisin expression in muscles and decreased MD2-TLR4 complex formation, and (iii) irisin disturbed MD2-TLR4 assembly by competitively binding with MD2. Together, the results suggest an antagonistic role of exercise-induced irisin in the TLR4 pathway in NAFLD mice.

The MD2-TLR4 pathway activates the MyD88-dependent MAPK and NF-κB pathways to induce pro-inflammatory cytokines [6], and has been regarded as one of the critical pathophysiological mechanisms of NAFLD [37,38]. Interestingly, exercise was shown to downregulate TLR4 expression in muscle and leukocytes in humans [39,40], and in muscle, vascular tissue, blood cells, and adipose tissue in mice [41]. Yang et al. also showed that exercise ameliorated TLR4 mRNA levels in NAFLD mice [42]. Similarly, our results showed that the mRNA levels of MD2 and TLR4 were both decreased by exercise. However, the protein levels of MD2 and TLR4 did not change. Instead, we discovered for the first time that exercise attenuated the activity of the TLR4 pathway by disrupting MD2-TLR4 assembly. Thus, regulation of the inflammatory response in NAFLD by exercise was at least partly mediated by irisin blocking the MD2-TLR4 pathway.

In an attempt to find the underlying mechanism, we focused on the role of myokines, which are believed to mediate the benefits of exercise [43]. Among the various myokines discovered to date [44], irisin is a myokine that plays multiple roles in liver disease. Irisin ameliorated endoplasmic reticulum (ER) stress and liver fibrosis in hepatic stellate cells [45], regulated cholesterol homeostasis [46,47], and improved fatty acid oxidation and glucose metabolism in mice [22,48]. These findings emphasize that irisin has the potential to mediate the protective effect of exercise against NAFLD development. In our study, irisin was induced by exercise in skeletal muscle. Among the muscle depots measured, the soleus muscle showed the highest basal irisin content, which indicates that the soleus could be a major source of circulating irisin. Consistent with our results, Roca-Rivada et al. reported that under basal conditions, soleus muscle secretes approximately 40% more FNDC5/irisin than gastrocnemius muscle [49]. Despite differences in muscle phenotypes, irisin content was induced by exercise in all three depots. Plasma irisin levels were also elevated by exercise, reflecting the increase in muscle.

Regarding its anti-inflammatory role, irisin was previously shown to protect against ischemia/reperfusion-induced brain injury and LPS-induced inflammation in macrophages by downregulating TLR4 and MyD88 expression [24,25]. A recent study also reported that irisin alleviates lipotoxicity-induced β-cell insulin resistance and inflammatory response through inhibition of TLR4/NF-kB pathways [50]. Consistently, we discovered that irisin inhibited MD2-TLR4 formation in the liver, indicating irisin as the effector for exercise in blocking the TLR4 pathway. While the above-mentioned reports observed changes in TLR4 mRNA and protein levels by irisin treatment, we identified through the pull-down assay, ELISA, and surface plasmon resonance analysis that irisin directly bound to MD2 but not TLR4. More importantly, irisin-bound MD2 showed a lower affinity for PA and LPS, albeit no change in affinity for TLR4, suggesting that irisin competitively bound to MD2 to inhibit LPS-MD2 or PA-MD2 formation. This phenomenon is similar for eritoran, a synthetic analog of LPS, which is a strong antagonist of the MD2-TLR4 complex that competitively binds to the hydrophobic pocket in human MD2 with no direct interaction between TLR4 [51]. Although we identified through simulation the potential motifs for MD2 that are likely to interact with irisin, crystallization and structural studies are needed to clarify the irisin-MD2-TLR4 complex. Notably, the crystal structure of irisin displayed a pattern of dimerization (PDB: 4LSD) [36], but we could not confirm whether this binary structure was necessary for the binding ability of irisin.

Recent studies have reported that chronic exercise can reduce circulating LPS levels, possibly through promoting gut barrier integrity [52]. However, the underlying mechanism remains unknown. Our results show that irisin interferes with the recognition of LPS by MD2, but whether irisin can also mediate the effect of exercise on regulating LPS levels is not elucidated. Given that LPS levels are elevated in NAFLD [53,54], it would be worth exploring the direct effect of irisin on systemic or portal LPS levels in further studies.

Although skeletal muscle is the predominant organ for irisin secretion [28], the local expression of irisin in other tissues has also been described [33,55]. In our study, hepatic irisin was increased in the HFD group, indicating a compensatory secretion under pathological conditions. The elevated levels remained after exercise training. Interestingly, this trend was consistent with the in vivo measurement of the irisin-MD2 complex content in the liver, which further implies the independent role of local irisin in the liver, in addition to the circulatory levels derived from muscle. Recently, Canivet et al. reported that hepatic expression of FNDC5 increased with hepatic steatosis and liver injury without impacting the systemic level of irisin in mice and humans [56]. They also observed increased steatosis, insulin resistance, and apoptosis in FNDC5-silenced hepatocytes, emphasizing on the local effect of irisin in the liver. To what extent local versus systemic irisin levels contribute to improving NAFLD is worth investigating in the future.

TLR4 is closely associated with the activation of the NLRP3 inflammasome, which leads to activation of caspase-1 and cleavage of GSDMD and pro-IL-1β/pro-IL-18, leading to the formation of pores in the cell membrane by the N-terminal fragment of GSDMD and the promotion of mature IL-1β/IL-18 release into the extracellular environment [57]. Studies have shown that the NLRP3 inflammasome contributed to hepatic inflammation [58,59], but whether irisin has an effect on this pathway remains unclear. Recent findings showed that irisin exerts cardioprotective effects by inhibiting NLRP3-mediated pyroptosis [60]. However, in our study, we found that only NLRP3 inflammasome priming was triggered in HFD mice and attenuated by exercise, whereas the activity of the NLRP3 inflammasome in the cleavage of caspase-1 and GSDMD was unaltered. Therefore, we ruled out the possible involvement of the NLRP3 inflammasome in our model.

In conclusion, our study provides evidence that irisin induced by exercise prevents inflammation in NAFLD by competitively binding with MD2 and highlights a new role of irisin as an antagonist of the TLR4 pathway, where the only known irisin receptor to date is the αV integrin receptor [23]. Although we utilized liver lysates to partly support the effect of irisin on the MD2-TLR4 complex in vivo, the therapeutic effect of irisin on NAFLD would need to be confirmed by injection of irisin in NAFLD mice, which remains a limitation of our study. With continued research, irisin could be developed into a high-performance preventive and therapeutic drug for NAFLD.

## Data Availability

The data presented in this study are available in the figures and tables of this manuscript.

## Supplementary Materials

The following are available online at https://www.mdpi.com/article/10.3390/cells10123306/s1, Table S1: Antibody list, Table S2: Mouse primers used for real-time qPCR assay, Figure S1: Whole-slide imaging of histological staining in liver tissues, Figure S2: HFD induces NLPR3 inflammasome assembly but not activation in mouse liver, Figure S3: The protein and mRNA change of MD2 and TLR4 in mouse liver, Figure S4: Densitometric quantifications of MAPK and NF-κB pathways in liver tissues and AML12 cells, Figure S5: Irisin blocks MAPK and NF-κB pathways to inhibit inflammation in macrophages.
