# Supplementary material for: Exercise-Induced Irisin Decreases Inflammation and Improves NAFLD by Competitive Binding with MD2
Source: Cells. 2021 Nov 25;10(12):3306. doi: 10.3390/cells10123306 (PMC8699279; doi:10.3390/cells10123306)
Supplement: Supplementary file 1 [file cells-10-03306-s001.zip › Supplementary data_FINAL.pdf]

Supplementary Table S1. Antibody list

Antibody	Source	Company	Cat No.
β-Tubulin	Rabbit	CST	2146
FABP4	Rabbit	CST	3544
COL1A1	Rabbit	CST	72026
F4/80	Rabbit	CST	70076
NLRP3	Rabbit	CST	15101
Caspase-1	Rabbit	CST	3866
Cleaved GSDMD	Rabbit	CST	50928
p-ERK1/2	Rabbit	CST	9101
ERK1/2	Rabbit	CST	9102
p-JNK	Rabbit	CST	9251
JNK	Rabbit	CST	9252
p38	Rabbit	CST	8690
p-p65	Rabbit	CST	3033
p65	Rabbit	CST	8242
PPARα	Mouse	Santa cruz	sc-398394
IL-6	Mouse	Santa cruz	sc-28343
TLR4	Mouse	Santa cruz	sc-293072
p-p38	Mouse	Santa cruz	sc-166182
IκB-α	Mouse	Santa cruz	sc-1643
TGFβ1	Rabbit	Abcam	ab92486
GSDMD	Rabbit	Abcam	ab155233
MD2	Rabbit	Abcam	ab24182
MD2	Mouse	Invitrogen	MA5-15766
Irisin (Biotin)	Rabbit	Adipogen	AG-25B-0027B

Supplementary Table S2. Mouse primers used for real-time qPCR assay

Gene	Forward sequence	Reverse sequence
Fabp4	AAGGTGAAGAGCATCATAACCCT	TCACGCCTTTCATAACACATTCC
Ppara	AGAGCCCCATCTGTCCTCTC	ACTGGTAGTCTGCAAAACCAAA
Tgfb1	CACCTGCAAGACCATCGACA	CATAGTAGTCCGCTTCGGGC
Col1	GCTCCTCTTAGGGGCCACT	CCACGTCTCACCATTGGGG
Il6	AGTTGCCTTCTTGGGACTGA	TCCACGATTTCCCAGAGAAC
Il1b	GCAACTGTTCCTGAACTCAACT	ATCTTTTGGGGTCCGTCAACT
Tnf	CCCTCACACTCAGATCATCTTCT	GCTACGACGTGGGCTACAG
Ccl2	CTTCTGGGCCTGCTGTTCA	CCAGCCTACTCATTGGGATCA
Icam1	CTGGGCTTGGAGACTCAGTG	CCACACTCTCCGGAAACGAA
Vcam1	AGTTGGGGATTCGGTTGTTCT	CCCCTCATTCCTTACCACCC
Md2	CGCTGCTTTCTCCCATATTGA	CCTCAGTCTTATGCAGGGTTCA
Tlr4	ATGGCATGGCTTACACCACC	GAGGCCAATTTTGTCTCCACA
18s	CGAAAGCATTTGCCAAGAAT	AGTCGGCATCGTTTATGGTC

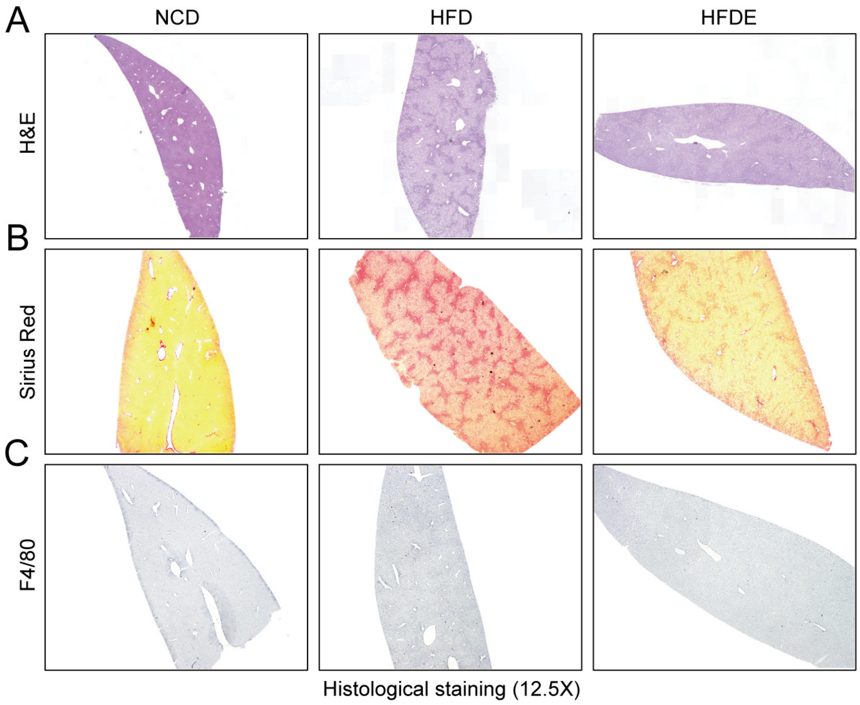

Supplementary Figure S1: Whole slide imagings of histological staining in liver tissues. (A) Representative images of liver sections stained by H&E (12.5X). (B) Representative images of liver sections stained by Sirius Red (12.5X). (C) Representative immunohistochemistry staining of liver sections with F4/80 (12.5X).

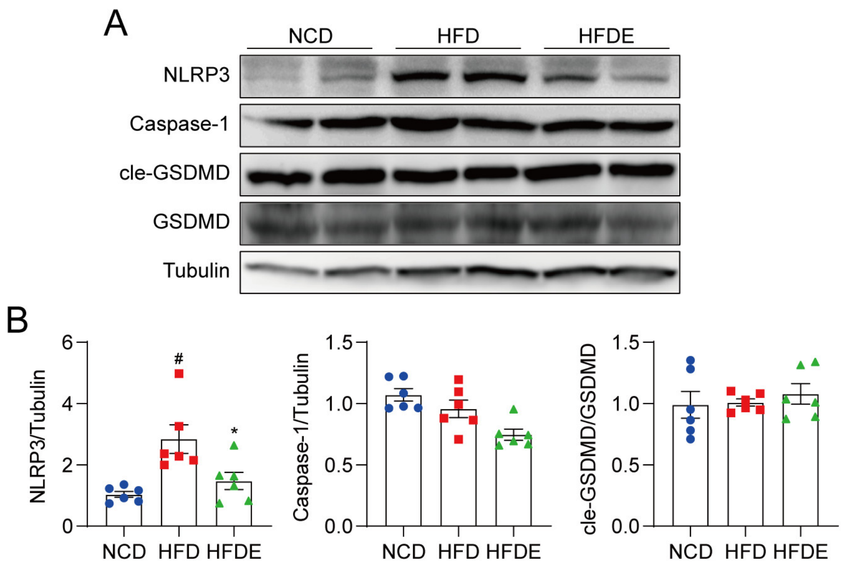

Supplementary Figure S2: HFD induces NLPR3 inflammasome assemble but not activation in mice liver. (A) Protein levels of NLRP3, Caspase-1, and cleaved-GSDMD in mice liver tissues, total GSDMD and tubulin were used as the loading control. (B) Densitometric quantifications for (A). Data were presented as mean ± SEM. $^{\#}$p vs. NCD group.*p < 0.05 vs. HFD group.

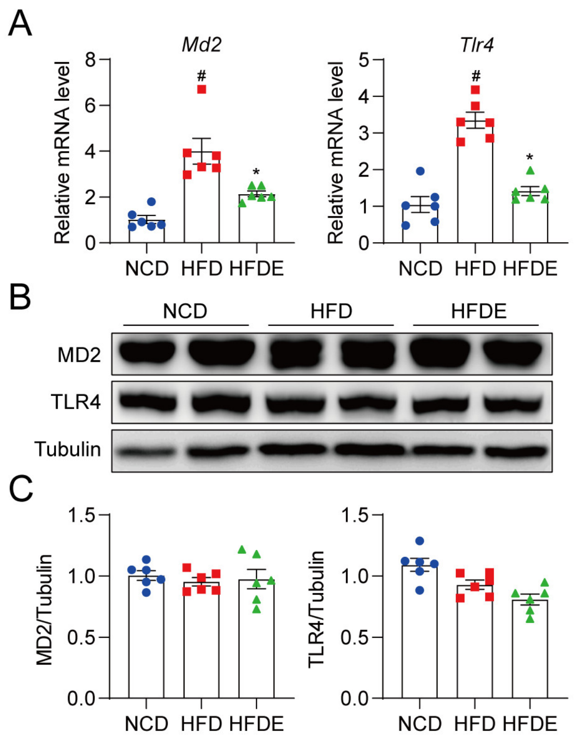

Supplementary Figure S3: The protein and mRNA change of MD2 and TLR4 in mice liver. (A) Relative mRNA levels of *Md2* and *Tlr4* in mice liver tissues. (B) Protein levels of MD2 and TLR4 in mice liver tissues, tubulin was used as the loading control. (C) Densitometric quantifications for (B). Data were presented as mean ± SEM. $^{\#}p < 0.05$ vs. NCD group. $^{*}p < 0.05$ vs. HFD group.

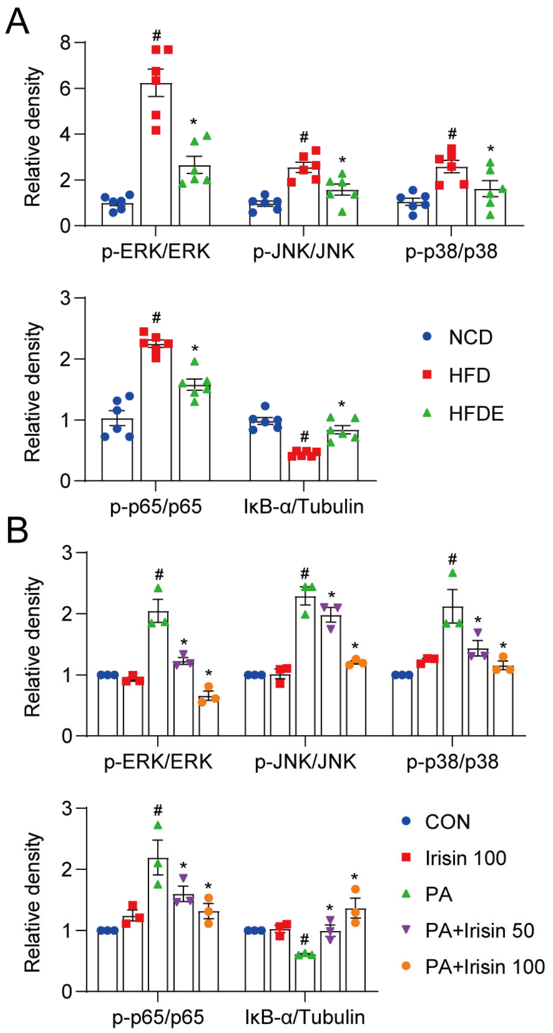

Supplementary Figure S4: Densitometric quantifications of MAPK and NF-κB pathways in liver tissues and AML12 cells. (A) Densitometric quantifications for Figure 4B. (B) Densitometric quantifications for Figure 7B. Data were presented as mean ± SEM. $^{\#}p < 0.05$ vs. NCD or CON group. $^{*}p < 0.05$ vs. HFD or PA group.

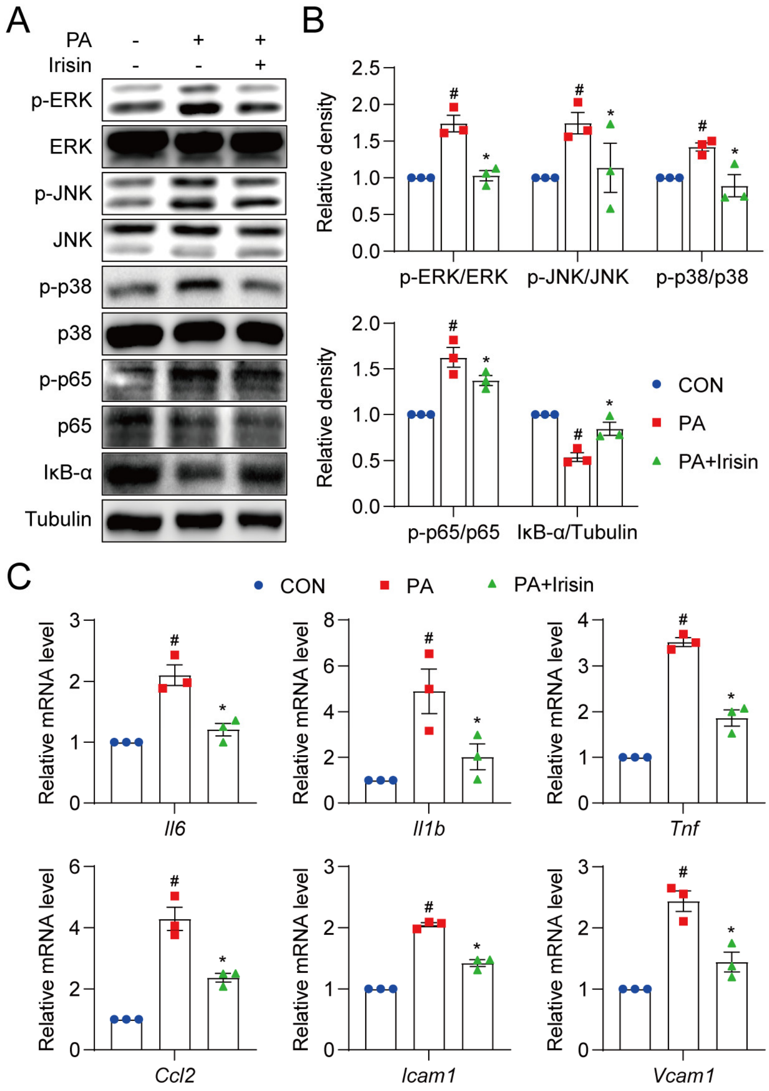

Supplementary Figure S5: Irisin blocks MAPK and NF-κB pathways to inhibit inflammation in macrophages. (A) RAW264.7 cells were pre-treated with recombinant irisin (100 ng/ml) for 30 min followed by exposure to 200 μM PA for 2 h. Protein levels of MAPK pathway and NF-κB pathway, including p-ERK, p-JNK, p-p38, p-p65, IκB-α were determined. Corresponded unphosphorylated proteins and tubulin were used as the loading control. (B) Densitometric quantifications for (A). (C) RAW264.7 cells were pre-treated with recombinant irisin (100 ng/ml) for 30 min followed by exposure to 200 μM PA for 12 h. Relative mRNA level of *Il6*, *Il1b*, *Tnf*, *Ccl2*, *Icam1*, and *Vcam1* were detected. Data were presented as mean ± SEM. $^{\#}p < 0.05$ vs. CON group. $^{*}p < 0.05$ vs. PA group.
